# Supplementary material for: Comparative genomics provides new insights into the diversity, physiology, and sexuality of the only industrially exploited tremellomycete: Phaffia rhodozyma
Source: BMC Genomics. 2016 Nov 9;17:901. doi: 10.1186/s12864-016-3244-7 (PMC5103461; doi:10.1186/s12864-016-3244-7)
Supplement: Additional file 6: — List of orphan genes with links to PFAM (related to Additional file 1: Table S1). (ZIP 1428 kb) [file 12864_2016_3244_MOESM6_ESM.zip › BLAST_HTML_FTR/G04325_P.html]

BLAST Search Results


```
BLASTP 2.2.27+


Reference:
Stephen F. Altschul, Thomas L. Madden, Alejandro A. Schäffer,
Jinghui Zhang, Zheng Zhang, Webb Miller, and David J. Lipman (1997),
"Gapped BLAST and PSI-BLAST: a new generation of protein database
search programs", Nucleic Acids Res. 25:3389-3402.


Reference for
composition-based statistics:
Alejandro A. Schäffer, L. Aravind, Thomas L. Madden, Sergei
Shavirin, John L. Spouge, Yuri I. Wolf, Eugene V. Koonin, and
Stephen F. Altschul (2001), "Improving the accuracy of PSI-BLAST
protein database searches with composition-based statistics and
other refinements", Nucleic Acids Res. 29:2994-3005.


Database: nr
           71,551,133 sequences; 26,053,659,533 total letters


Query= G04325_P

Length=427
                                                                      Score     E
Sequences producing significant alignments:                          (Bits)  Value

emb|CDZ96585.1|  hypothetical protein [Xanthophyllomyces dendrorh...   843    0.0  
emb|CDZ96324.1|  hypothetical protein [Xanthophyllomyces dendrorh...  70.5    5e-10


 >emb|CDZ96585.1| hypothetical protein [Xanthophyllomyces dendrorhous]
Length=426

 Score =  843 bits (2179),  Expect = 0.0, Method: Compositional matrix adjust.
 Identities = 426/426 (100%), Positives = 426/426 (100%), Gaps = 0/426 (0%)

Query  1    MSVVPSNHGSHRGAFNSKPNTHQHSHTLNSSFRTASASPRPVLRNTINPSVQAVVPSSGN  60
            MSVVPSNHGSHRGAFNSKPNTHQHSHTLNSSFRTASASPRPVLRNTINPSVQAVVPSSGN
Sbjct  1    MSVVPSNHGSHRGAFNSKPNTHQHSHTLNSSFRTASASPRPVLRNTINPSVQAVVPSSGN  60

Query  61   TTSTNTTAAATTTHPSKPARQSHKSTPVKGTTATTVVAASQIPGLLTLSKPLEDKFASRP  120
            TTSTNTTAAATTTHPSKPARQSHKSTPVKGTTATTVVAASQIPGLLTLSKPLEDKFASRP
Sbjct  61   TTSTNTTAAATTTHPSKPARQSHKSTPVKGTTATTVVAASQIPGLLTLSKPLEDKFASRP  120

Query  121  APKKNHSHKHQNFAQRTTGTDNSAVDPSIKVASDKDVPSKSPRRSRHKRTKPDGNPPGPT  180
            APKKNHSHKHQNFAQRTTGTDNSAVDPSIKVASDKDVPSKSPRRSRHKRTKPDGNPPGPT
Sbjct  121  APKKNHSHKHQNFAQRTTGTDNSAVDPSIKVASDKDVPSKSPRRSRHKRTKPDGNPPGPT  180

Query  181  VDVPHASIPGVTEPVHKRRPKASSNQRSASRISAGRARVRPMKEESEEDESEEWEMPLEK  240
            VDVPHASIPGVTEPVHKRRPKASSNQRSASRISAGRARVRPMKEESEEDESEEWEMPLEK
Sbjct  181  VDVPHASIPGVTEPVHKRRPKASSNQRSASRISAGRARVRPMKEESEEDESEEWEMPLEK  240

Query  241  RGTDVLTWQQTSPPSAPTKPTPKMPSPRAQVNRLQTRSISPSPAVSISTPPSATNLTWQQ  300
            RGTDVLTWQQTSPPSAPTKPTPKMPSPRAQVNRLQTRSISPSPAVSISTPPSATNLTWQQ
Sbjct  241  RGTDVLTWQQTSPPSAPTKPTPKMPSPRAQVNRLQTRSISPSPAVSISTPPSATNLTWQQ  300

Query  301  ALFESSAPVVVSKKKNPTANLQRRSSFESSLSSSAPPAQFAGPTSSTATRPAKGNTFPRA  360
            ALFESSAPVVVSKKKNPTANLQRRSSFESSLSSSAPPAQFAGPTSSTATRPAKGNTFPRA
Sbjct  301  ALFESSAPVVVSKKKNPTANLQRRSSFESSLSSSAPPAQFAGPTSSTATRPAKGNTFPRA  360

Query  361  HHLTQNNQRASSGHAKSISLSTLGLAGGSIESGADNSAKGPLYAGSVFQNSPLAGDLPKP  420
            HHLTQNNQRASSGHAKSISLSTLGLAGGSIESGADNSAKGPLYAGSVFQNSPLAGDLPKP
Sbjct  361  HHLTQNNQRASSGHAKSISLSTLGLAGGSIESGADNSAKGPLYAGSVFQNSPLAGDLPKP  420

Query  421  RFGLKI  426
            RFGLKI
Sbjct  421  RFGLKI  426


>emb|CDZ96324.1| hypothetical protein [Xanthophyllomyces dendrorhous]
Length=332

 Score = 70.5 bits (171),  Expect = 5e-10, Method: Compositional matrix adjust.
 Identities = 71/220 (32%), Positives = 103/220 (47%), Gaps = 39/220 (18%)

Query  230  ESEEWEMPLEKRGTDVLTWQQTSPPSAPTKPTPKMP--SPRAQVNRLQTRSISPSPAVSI  287
            E++ W+MP++  G DVLTWQQTS  S+P+K        + R+Q  +  + S S +P    
Sbjct  127  EADVWDMPIKASGRDVLTWQQTSSSSSPSKLPSSPRPRAARSQQGQPVSTSKSIAPFSRS  186

Query  288  STPPSA-----------------TNLTWQQALFESSAPVVVSKKKNPTANLQRRSSFESS  330
            S  P A                 +NLTWQQALFE++ P     K      ++RR+S  + 
Sbjct  187  SNQPLAGPFHHASSSSITAVSSNSNLTWQQALFETAHPASSRPK------IRRRASLGNE  240

Query  331  LSSSAPPAQFAGPTSSTATRPAKGNTFPRAHHLTQNNQRASS---GHAKSISLSTLGLAG  387
              S   P             P K N+    H  +  N+R S     + + ++ S+     
Sbjct  241  --SGTAPVHLP-----RLGVPVKSNSI--HHSSSMKNKRVSGSPPNNGERVNGSSYPAGT  291

Query  388  GSIESGADNSAKGPL--YAGSVFQNSPLAGDLPKPRFGLK  425
               +SG     +G +  YAG  FQN+PLAGDLPKPRFG +
Sbjct  292  VGSDSGGVRRPEGEINTYAGGTFQNAPLAGDLPKPRFGAR  331


Lambda      K        H        a         alpha
   0.307    0.121    0.346    0.792     4.96 

Gapped
Lambda      K        H        a         alpha    sigma
   0.267   0.0410    0.140     1.90     42.6     43.6 

Effective search space used: 4139136114416


  Database: nr
    Posted date:  Sep 23, 2015 12:05 AM
  Number of letters in database: 26,053,659,533
  Number of sequences in database:  71,551,133


Matrix: BLOSUM62
Gap Penalties: Existence: 11, Extension: 1
Neighboring words threshold: 11
Window for multiple hits: 40
```
